# Case of Cartilaginous Exostosis of the Gondyle, Etc.

**Published:** 1853-12

**Authors:** Daniel Brainard

**Affiliations:** Professor of Surgery in Rush Medical College; President of the Illinois State Medical Society, etc.; Chicago


From the American Journal of the Medical Sciences.

*Case of Cartilaginous Exostosis of the Condyle, Ramus, and Angle of the Lower Jaw, for which Resection with Removal of the Parotid Gland and Zygomatic Arch was successfully performed.* By DANIEL BRAINARD, M. D., Professor of Surgery in Rush Medical College; President of the Illinois State Medical Society, etc.

W. S., æt. 32 years, formerly of good constitution, first perceived, in October, 1850, that the movements of the lower jaw were imperfect. Soon after, he noticed a swelling at the left side of the articulation. It was hard, unattended with pain or discoloration, and increased slowly. In February, 1852, an abscess formed upon the side of the neck, below the angle of the jaw, from which, when opened, a fistulous passage leading to the bone remained. Some months afterwards, another abscess formed in front of the ear, from which pus and saliva were discharged.—Another formed upon the zygoma. During the whole of this time, the patient's health was not materially impaired.

He was treated principally with different preparations of iodine, internally and externally.

*Present State*, *Jan.* 19, 1853. There is a hard tumour, extending from the temple above below the angle of the jaw. and from the middle of the cheek to the ear. It is very prominent and irregular, and has on its surface three fistulous openings, corresponding to the situation of the abscesses. They were found to converge toward the articulation. The mouth could be but slightly opened, but enough to enable me to perceive that the tumour encroached upon the fauces and pharynx. There is pain most severe at night. When he eats, the saliva flows freely from the middle fistula. His health is good, no sallowness, and only a little emaciation, which dates from about a month.

Operation performed Jan. 27, 1853, with the assistance of Drs. Herrick, Freer, and Morfit, as follows:

The patient having been placed under the effect of chloroform, a curved incision was made, commencing an inch above the zygoma, carried down in front of the ear over the angle of the jaw, and brought forward as far as the side of the chin. The flap was raised, and the integuments dissected up from the surface of the tumour behind.

The bicuspid tooth was extracted, the tissues detached from the jaw, and the bone divided with a chain-saw.

At this stage of the operation, it was necessary to allow the effects of the chloroform to pass off, as the flow of blood into the mouth and throat rendered it necessary that it should be constantly ejected.

The separation of the tumour below and within was next effected, as far back as the mastoid process, against which it was placed.

An attempt was now made to turn it outwards, but it was still almost immoveable, and it was necessary to attack it from above.

The temporal muscle was divided, the zygomatic arch cut away at its anterior and posterior extremities, the massete muscle divided, and a chisel and mallet used to separate the articulation; but the bones were so confounded together, that a part of the glenoid cavity was cut away. A lever being inserted behind the tumour, strong efforts were made to turn it forward, but without success. It was then attacked from within; the pterygoid muscles, which were semicartilaginous, were divided at their attachment to the pterygoid processes. The mass was then raised from its position, and its remaining adhesion separated.

The vessels which gave blood were very numerous. The facial, lingual, external, carotid and numerous smaller branches, required the ligature.

The ninth pair of nerves, the portio dura, and the dental branch of the lingual were divided.

On examining the wound, it was found to lay bare the cornu of the os hyoides, the mastoid and styloid processes, and the transverse processes of the cervical vertebræ. The internal jugular vein and the internal carotid artery were seen at the bottom of the wound. The temporal fossa was excavated, the muscles of the tongue and pharynx laid bare. The parotid gland had been partially obliterated by pressure, and no trace of it now remained.

The wound was brought together by stitches, and cold-water dressings applied. A full dose of opium was administered. The operation, which lasted an hour, was well borne.

After treatment, no symptoms requiring especial notice occurred. The ligatures were all removed by the tenth day. The wound had adhered throughout, excepting at the point where the ligatures prevented.

*Dec.* 5. The patient's health is good. He can sit up and walk about, but requires an anodyne at night, on account of pain.

*July* 17. There still remains a fistulous opening, leading to the situation of the glenoid cavity, through which a small piece of bone has been discharged. The lower jaw has fallen so much to one side that the patient cannot masticate. He is emaciated, and appears to be suffering from inanition, resulting from insufficient nourishment. He still requires anodynes to procure rest at night. There is no appearance of return of the disease.

On examination of the tumour, the bone seemed greatly enlarged in every sense, the ramus of the jaw and the angle were four times their natural thickness, the condyle partially absorbed and altered in shape. This mass, which had the appearance of bone, and from which the periosteum could readily be separated, was so soft as to be easily transfixed with a sharp instrument. The surrounding tissues were infiltrated with serum and had an appearance like firm jelly, but, after maceration, presented no appearance of morbid deposit.

Chicago, August 1, 1853.

